# Case report: Prenatal diagnosis of fetal intracranial hemorrhage due to compound mutations in the *JAM3* gene

**DOI:** 10.3389/fgene.2022.1036231

**Published:** 2022-10-19

**Authors:** Min Xu, Pengzhen Jin, Yingzhi Huang, Yeqing Qian, Miaochun Lin, Juan Zuo, Jin Zhu, Zhaohui Li, Minyue Dong

**Affiliations:** ^1^ Laboratory of Prenatal Diagnosis, Mindong Hospital Affiliated to Fujian Medical University, Ningde, China; ^2^ Women’s Hospital, School of Medicine, Zhejiang University, Hangzhou, China; ^3^ Key Laboratory of Reproductive Genetics, Ministry of Education, Hangzhou, China

**Keywords:** intracranial hemorrhage, whole-exome sequencing, *JAM3*, prenatal diagnosis, genetic counseling

## Abstract

Intracranial hemorrhage is a common complication in preterm infants but occasionally occurs in fetuses. Disruptions of the genes, such as the *COL4A1* and *COL4A2* genes*,* are common genetic causes identified in fetal intracranial hemorrhage; however, the disruptions of the *JAM3* gene are rarely reported. In the current investigation, fetal intracranial hemorrhage and dilated lateral ventricles were observed in three consecutive siblings in a pedigree. The pregnancies were terminated, and whole-exome sequencing, followed by Sanger sequencing, was performed on the affected fetuses. Pre-implantation genetic testing for monogenic diseases was performed to avoid the recurrence. The compound heterozygous variants of c.712 + 2T > A and c.813C > G p.Tyr271* in the *JAM3* gene (NM_032801.4) were identified in the proband and its affected brother, which were predicted to be pathogenic. The variant of c.813C > G p.Tyr271* but not c.712 + 2T > A was identified in the fourth fetus, implying a good prognosis. Our findings expanded the spectrum of the pathogenic mutations in the *JAM3* gene and revealed an important application of fetal whole-exome sequencing in idiopathic fetal intracranial hemorrhage.

## Introduction

Intracranial hemorrhage (ICH) is defined as non-traumatic bleeding within the cranium (de Oliveira et al., 2016; Kutuk et al., 2014). It is a complication in preterm neonates and occasionally occurs in preterm fetuses with an incidence ranging from 1/1,00,000 to 1/1,000 (Cavaliere et al., 2021; Kutuk et al., 2014). Fetal ICH is usually associated with cerebral palsy, neurodevelopmental delay, and other adverse impacts (Cavaliere et al., 2021; Kutuk et al., 2014), increasing the risk of perinatal mortality and morbidity (Sileo et al., 2022). Advanced maternal age, hypertension, preeclampsia or eclampsia, coagulopathy, alloimmune thrombocytopenia, twin-to-twin transfusion syndrome, and infectious diseases are known causes leading to fetal ICH (Hausman-Kedem et al., 2021; Meeks et al., 2020). However, nearly 75% of the cases have no identifiable risk factors (Armstrong-Wells et al., 2009).

Recently, a number of pathogenic mutations were detected in idiopathic ICH, including the *COL4A1*, *COL4A2*, *F11*, *F7*, *FGA*, *VWF*, and *GP1BA* genes (Cavaliere et al., 2021; Hausman-Kedem et al., 2021; Lichtenbelt et al., 2012; Vermeulen et al., 2011). Observations about the disruption of the *JAM3* gene leading to ICH are currently available. ICH, caused by pathogenic mutations in the *JAM3* gene, has been reported in three publications (Akawi et al., 2013; De Rose et al., 2021; Mochida et al., 2010), few of which were diagnosed prenatally.

Patients with ICH caused by *JAM3* mutations usually die in infancy (Akawi et al., 2013; De Rose et al., 2021; Mochida et al., 2010). Therefore, the prenatal identification of genetic causes is necessary for therapeutic decision-making. Whole-exome sequencing (WES) is recommended as a routine test when severe structure abnormalities are newly diagnosed (Jin et al., 2020).

Herein, we described a pedigree with three consecutive fetal ICH and dilated lateral ventricles. WES, followed by Sanger sequencing, was performed on the proband (II 2) and his affected brother (II 3). The compound heterozygous variants of c.712 + 2T > A and c.813C > G were identified in the *JAM3* gene, which were predicted to be pathogenic. Then, pre-implantation genetic testing for monogenic diseases (PGT-M) was performed. The variant of c.813C > G p.Tyr271* but not c.712 + 2T > A was identified in the fourth fetus, implying a good pregnancy outcome. The current investigation expands the mutation spectrum of the *JAM3* gene, and the combination of ultrasound and fetal whole-exome sequencing is an effective method for the prenatal diagnosis of fetal ICH.

## Subjects and methods

### Case presentation

The 33-year-old non-consanguineous healthy couple conceived three fetuses with ICH. No obvious risk factors such as hypertension, coagulopathy, alloimmune thrombocytopenia, and infection were found during the pregnancy.

**FIGURE 1:**
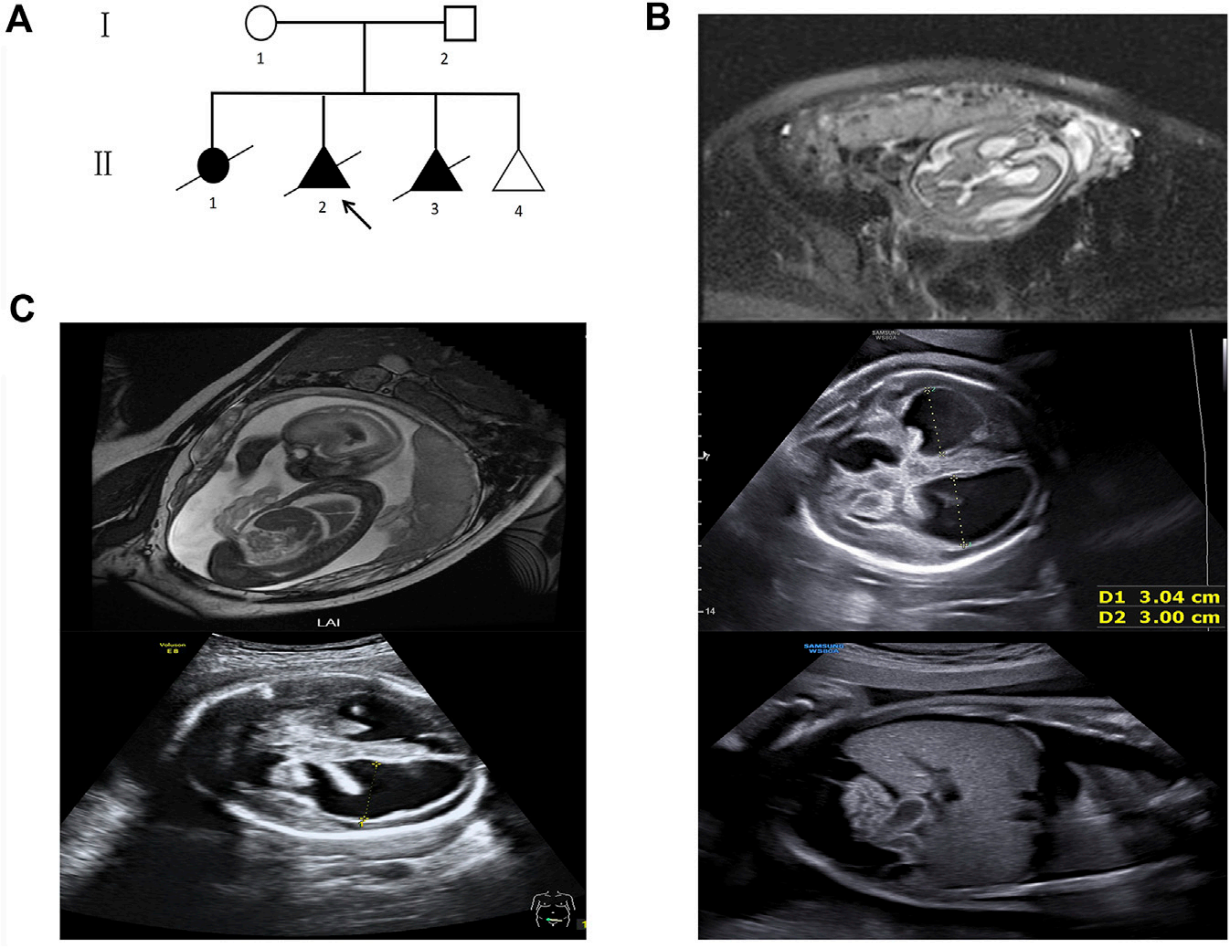
Pedigree and fetal ultrasound scan and MRI findings. **(A)** Pedigree of the family. The arrow refers to the proband. The solid circle (female) and triangle (fetus) represent the affected siblings; **(B)** ultrasound and MR imaging in the fetus (II 2): the enlarged bilateral ventricles, intraventricular hemorrhage and severe hydrocephalus, the infantile edema of the skin, bilateral pleural effusion, and ascites in the fetus; **(C)** ultrasound and MR imaging in the fetus (II 3): the enlarged bilateral ventricles, intraventricular hemorrhage, and severe hydrocephalus in the fetus.

Their first female child (II 1) was delivered by emergency cesarean section at the 34th week of gestation (Figure 1A). Severe dilated, bilateral ventricles were noticed, and she died in the neonatal period. Unfortunately, no genetic tests were performed and no biological samples were available.

The second fetus (II 2) was conceived naturally. Fetal cranial magnetic resonance imaging (MRI) at the 29th week revealed hydrocephalus and dilated ventricles (Figure 1B). The ultrasound scan at the 31st week showed severe cerebral ventriculomegaly measuring 30 mm, edema of the skin, bilateral pleural effusion, and ascites in the fetus (Figure 1B). The pregnancy was terminated after genetic counseling. Then, a chromosomal microarray assay (CMA) was provided, but no duplication or deletion was identified (Supplementary Material).

The third fetus (II 3) was also naturally conceived. Fetal cranial MRI was performed at the 24th week, and dilated ventricles and intraventricular hemorrhage were observed (Figure 1C). The ultrasound scan at the 29th week showed severe cerebral ventriculomegaly measuring 28 mm. Bright echogenic linings were described in the bilateral choroid plexus (Figure 1C). The pregnancy was terminated after genetic counseling, and samples were taken for genetic testing.

The fourth conception was conceived *via* assisted reproductive technology (ART) with PGT-M. The mother came to our clinic for genetic counseling and received prenatal diagnosis at the 18th week of gestation.

The use of data and medical record was approved by the Ethics Committee of Women’s Hospital, School of Medicine Zhejiang University, and conformed to the Declaration of Helsinki (IRB-20220222-R). All participants provided their written informed consent.

### Amniocentesis and fetal karyotyping

Amniocentesis was performed at the 18th week as usual. A measure of 20 ml of the amniotic fluid, after discarding the first 2 ml, was taken for karyotyping or DNA extraction under real-time sonographic guidance as a routine. Amniotic fluid cells were cultured, and G-banding karyotyping was conducted on metaphase preparations with a targeted 400-band level using the GSL-120 CytoVision platform (Leica, Germany). The karyotypes were described according to the International System for Human Cytogenetic Nomenclature (Stevens-Kroef et al., 2017).

### Chromosomal microarray assay

Genomic DNA was extracted using the QIAamp DNA Mini Kit (QIAGEN, Duesseldorf, Germany), and then, chromosomal microarray assay (CMA) was performed using CytoScanTM HD arrays (Affymetrix, Santa Clara, CA, United States). Chromosome Analysis Suite (Thermo Fisher Scientific, ChAS) software was used to analyze the molecular karyotype. Copy number variations were interpreted according to the standards and guidelines of the American College of Medical Genetics (ACMG) (South et al., 2013).

### Whole-exome sequencing

Whole-exome sequencing (WES) was performed on the Illumina HiSeq2000 platform (Illumina, San Diego, CA, United States), with variants filtered through online population databases. The variants were subsequently annotated by multiple databases, including ClinVar (https://www.ncbi.nlm.nih.gov/clinvar/), gnomAD (http://gnomad.broadinstitute.org/), OMIM (https://www.omim.org/), ExAC (http://exac.broadinstitute.org/), HGMD (http://www.hgmd.cf.ac.uk/ac/index.php), and Leiden Open Variation Database (LOVD: http://www.dmd.nl/), and only variants with an allele frequency ≤1% were selected. To predict the pathogenic potential of the variant, several software programs including SpliceAI (https://spliceailookup.broadinstitute.org/), NetGene2 (https://services.healthtech.dtu.dk/service.php?NetGene2-2.42), and NNSplice (http://www.fruitfly.org/seq_tools/splice.html) were used. Finally, these variants were interpreted and referred to the American College of Medical Genetics and Genomics (ACMG) (Richards et al., 2015).

### Sanger sequencing

Sanger sequencing was carried out to validate the variants with an ABI 3130 DNA analyzer (Applied Biosystems™). The forward primer (5′-GTC​AGG​GAG​GAA​CAT​GCA​CAG​T-3′) and reverse primer (5′-CGG​AAG​AGT​TCT​CTA​AGC​TGA​TG-3′), and forward primer (5′-GTT​CTA​GGC​TAG​AAG​GAT​TGT​AAG-3′) and reverse primer (5′-CTC​AGG​AGC​TGC​ACA​ATC​ACT​C-3′) were used to amplify the PCR products in the *JAM3* gene. The procedure of the PCR was as follows: 95°C for 10 min, then followed by 35 cycles of 60°C for 30 s and 72°C for 1 min, and then 72°C for 10 min. The reaction was kept at 16°C.

## Results

### Identification of compound heterozygous variants

The compound variants of c.712 + 2T > A and c.813C > G p.Tyr271* were identified in the *JAM3* (NM_032801.4) gene, both in the proband (II 2) and his brother (II 3), neither of which were recorded in the gnomAD exome database, the Clinvar database, or the HGMD database (PM2). Segregation was observed in the family (PP1). The variant of c.712 + 2T > A, with a single nucleotide change at the canonical splicing sites, was predicted to impair the protein function (PSV1). It was predicted to cause abnormal splicing by SpliceAI software (0.98) and NetGene2 (PP3). The variant of c.813C > G p.Tyr271* generated non-sense-mediated premature termination codons, producing dominant-negative truncated proteins, leading to the deficiency of the protein (PSV1).According to the ACMG guidelines (Richards et al., 2015), both of the mutations were classified as pathogenic.

### Confirmation of the candidate mutations

**FIGURE 2:**
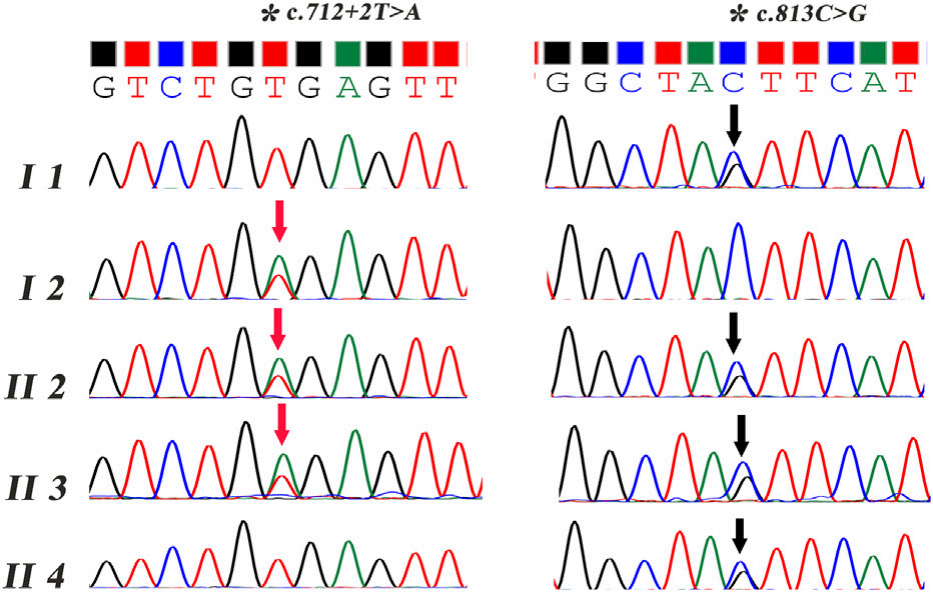
Sanger sequencing of the compound heterozygous variants in the *JAM3* gene. The red arrow refers to the variant of c.712 + 2T > A. The black arrow refers to the variant of c.813C > G.

Two primer sets were designed, and Sanger sequencing was performed on all the subjects except the first child (II 1). As shown in Figure 2, the mutations of c.813C > G p.Tyr271* and c.712 + 2T > A were confirmed both in the proband and his affected brother, which were inherited from his mother (I 1) and father (I 2), respectively.

### Prenatal diagnosis and follow-up

Karyotyping was normal, and no duplication or deletion was detected in the fourth fetus. The variant of c.813C > G p.Tyr271*, but not c.712 + 2T > A, was identified, which was consistent with the results of PGT-M. No structural abnormalities were observed during the pregnancy.

## Discussion

In the current investigation, WES, followed by Sanger sequencing, was performed to identify the pathogenic variants in a pedigree with the recurrent occurrence of fetal ICH and dilated lateral ventricles, and compound heterozygous variants of c.712 + 2T > A and c.813C > G were detected in the *JAM3* gene. Pre-implantation genetic testing for monogenic disease (PGT-M) was applied to avoid the inheritance of the disease.

Generally, ICH can be classified into five types, according to its anatomical location: intraventricular hemorrhage (IVH), subarachnoid hemorrhage (SAH), cerebellar hemorrhage, subdural hemorrhage, and intraparenchymal hemorrhage (Elchalal et al., 2005). IVH and cerebellar hemorrhage are more common in fetuses than in the other individuals (Hausman-Kedem et al., 2021). Advanced maternal age, hypertension, preeclampsia or eclampsia, coagulopathy, alloimmune thrombocytopenia, twin-to-twin transfusion syndrome, and infectious disease are independently associated with fetal ICH (Hausman-Kedem et al., 2021; Meeks et al., 2020). However, up to 75% of the cases have no identifiable risk factors (Armstrong-Wells et al., 2009) and are therefore classified as idiopathic (Cavaliere et al., 2021).

Recently, an increasing number of publications have disclosed the genetic causes identified in idiopathic ICH, and the known pathogenic variants include the following: *COL4A1*, *COL4A2*, *F11*, *F7*, *FGA*, *VWF*, *GP1BA*, and X-linked *GATA1* gene mutations (Cavaliere et al., 2021; Hausman-Kedem et al., 2021). Apparently, genetic causes are greatly underestimated for the most part, and the identification of the genetic causes is essential to determine prognosis, estimate the recurrence risk, and guide therapeutic decision-making. Recently, observations about disruptions of the *JAM3* gene leading to ICH have attracted the attention of researchers. To date, mutations in the *JAM3* gene have been identified in five unrelated families (Table 1).

**TABLE 1:** Summary of clinical and genetic features of mutations in the *JAM3* gene leading to fetal ICH.

Study		Clinical manifestations	Cranial MRI or CT	Genetic feature (*JAM3*)	Pregnancy outcome
Mochida et al. (2010)	VIII-21	Bilateral congenital cataracts, hepatomegaly, and bilateral cystic dysplasia	Multifocal intraparenchymal hemorrhage and liquefaction	Homozygous mutation intron 5 (c.747 + 1G > T)	Died a few minutes after birth
	VIII-22	Bilateral congenital cataracts, microphthalmia, small phallus, and undescended testes	Multifocal intraparenchymal hemorrhage and liquefaction	Homozygous mutation intron 5 (c.747 + 1G > T)	Died at the age of 3 years 4 months
	VIII-10	Bilateral congenital cataracts, systolic heart, murmur hepatomegaly, and thrombocytopenia	Multifocal intraparenchymal hemorrhage and liquefaction	Homozygous mutation intron 5 (c.747 + 1G > T)	Died at the age of 1 year 2 months
	VIII-29	Bilateral congenital cataracts, malrotated left kidney in the iliac fossa	Porencephalic cyst, enlarged lateral ventricles, and reduced white matter volume	Homozygous mutation intron 5 (c.747 + 1G > T)	Died at the age of 6 years 1 month
Akawi et al. (2013)	Family 1−Ⅱ2	Hemorrhagic hydrocephalus, bilateral nuclear cataracts, seizures, slopping forehead, bulging anterior fontanelle, and bitemporal grooving	Prominent expansive bleeding, calcification of the subependymal region, and multifocal intraparenchymal hemorrhage	Homozygous mutation exon 6 c.656G > A (p.C219Y)	Died at the age of 2 months
	Family 2−Ⅱ1	Bilateral cataracts, neurological deterioration, vomiting, and irritability	Intraventricular hemorrhage (grade IV), severe ventricular dilatation, and white matter abnormalities in both cerebral hemispheres and multiple subependymal cysts	Homozygous mutation exon 4 c.346G > A (p.E116K)	Died at the age of 2 weeks
	Family 3−Ⅱ5	Bilateral cataracts, irritability, and a bulging anterior fontanelle	Multifocal intraparenchymal hemorrhage, subependymal calcification, diffusely hypodense brain parenchyma, edema and cystic changes of the brain parenchyma, dilated ventricles, and a thin corpus callosum	Homozygous mutation c.2T > G (p.M1R)	Died at the age of 39 days
De Rose et al. (2021)		Bilateral irritable, excessive startle, hypotonia, apnea, and status epilepticus	Hemorrhage, multiple periventricular calcifications, porencephalic cysts, and hypoplasia of the cerebellar vermis	Homozygous mutation exon 6 c.690T > G (p.Cys230Trp)	Seizure was controlled, and the infant was discharged from the NICU

The *JAM3* gene is located on chromosome 11q25, with 930 bases in the open-reading frame, revealing 53% identity with *JAM2* or *JAM1* (Arrate et al., 2001; Luissint et al., 2014). It encodes junctional adhesion molecule 3 (JAM3), the third member of the JAM family, with three closely related proteins including JAM1 (JAM-A), JAM2 (JAM-B), and JAM3 (JAM-C) (Arrate et al., 2001; Langer et al., 2011; Mochida et al., 2010). JAM3 is widely distributed in the brain, bone marrow, heart, lung, liver, kidney, spleen, and testis nerve (Mochida et al., 2010), which functions through maintaining vascular homeostasis and barrier permeability, regulating cell adhesion and polarization (Gliki et al., 2004), and inducing self-renewal (Akawi et al., 2013; Luissint et al., 2014; Mandell and Parkos., 2005). Therefore, the absence of JAM3 is usually accompanied with growth retardation (Imhof et al., 2007), nuclear cataracts (De Rose et al., 2021), seizures (Mochida et al., 2010), and defects in multiple organ systems. Occasionally, hemorrhagic destruction of the brain and hydrocephalus is observed in the patients with the absence of JAM3 (Akawi et al., 2013; De Rose et al., 2021; Mochida et al., 2010).

Generally, JAM3 is distributed diversely between species. In humans, JAM3 is expressed extensively in the brain (Arrate et al., 2001), whereas the expression of Jam3 in the brain of adult mice remains at a low level (Mochida et al., 2010). Therefore, Jam3 was described to be absent in the mouse brain in some studies (Gliki et al., 2004; Mochida et al., 2010). Wyss et al. (2012) backcrossed JAM-C^−/−^ mice into the C57BL/6 background. They found that JAM-C^−/−^ C57BL/6 mice manifested hemorrhage, hydrocephalus, and enlarged lateral ventricles, providing a valuable model for the human-specific manifestations caused by the absence of JAM3.

In a consanguineous pedigree from the United Arab Emirates, eight individuals were characterized by intracranial hemorrhage, subependymal calcification, and congenital cataracts, four of who died in infancy. Among the survivors, seizures, generalized spasticity, and renal abnormalities were observed. Finally, a homozygous variant of c.747 + 1G > T was identified in the *JAM3* gene in this pedigree, uncovering their pathogenicity. It is heartbreaking to witness such an enormous number of sufferers in a family; therefore, the early detection and diagnosis is valuable in a pedigree when the proband is noticed.

In this investigation, intracranial hemorrhage and hydrocephalus were observed in consecutive siblings. The compound heterozygous variants of c.712 + 2T > A and c.813C > G were identified in the *JAM3* gene both in the proband and his affected brother, which were predicted to be pathogenic. To avoid the recurrence of the identical symptoms in the next conception, PGT-M was carried out after the genetic counseling.

PGT-M means identifying monogenic diseases by performing a genetic test before the embryo is implanted (Cimadomo et al., 2020). Only those unaffected embryos can be transferred (Traeger-Synodinos, 2006), which avoid the misery of affected pregnancies (He et al., 2019; Natsuaki and Dimler, 2018). It is an ideal method to improve pregnancy outcomes when monogenic diseases are confirmed in the family. In addition, severe fetal ICH, especially occurring in consecutive siblings, usually accompanies with a specific genetic cause. Therefore, the prenatal diagnosis is of great significance to guide therapeutic decision-making, which needs to be performed as early as possible.

## Conclusion

In conclusion, our investigation identified the compound heterozygous variants of c.712 + 2T > A and c.813C > G in the *JAM3* gene, expanding its spectrum. When recurrent occurrence of fetal ICH or other severe structure abnormalities are observed in a pedigree, fetal WES should be considered as routine genetic testing, and the combination of ultrasound and genetic testing might favor the prenatal diagnosis. In addition, the application of PGT-M provides an effective way to terminate the recurrent occurrence of monogenic diseases.

## Data Availability

The datasets for this article are not publicly available due to concerns regarding participant/patient anonymity. Requests to access the datasets should be directed to the corresponding authors.
