# Supplementary figures and images for: Case report: Prenatal diagnosis of fetal intracranial hemorrhage due to compound mutations in the JAM3 gene
Source: Front Genet. 2022 Oct 19;13:1036231. doi: 10.3389/fgene.2022.1036231 (PMC9629614; doi:10.3389/fgene.2022.1036231)

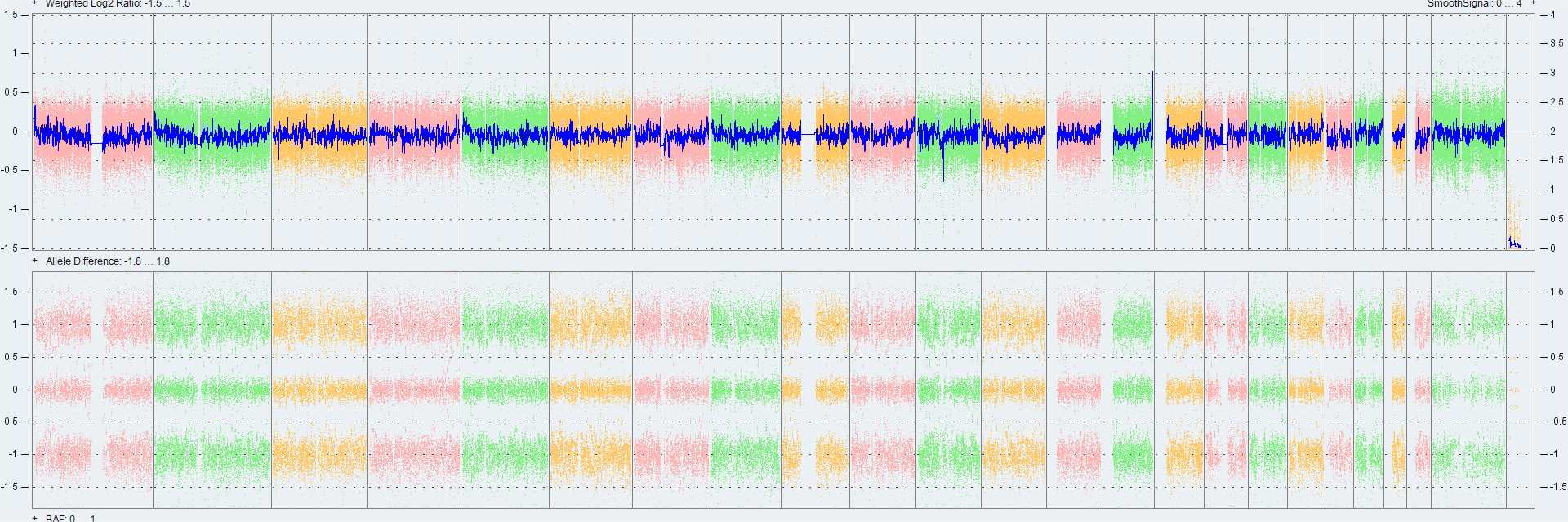

Supplement: Supplementary file 1 [file DataSheet1.ZIP › Supplementary Materials/Figure1 CMA in II2.png]

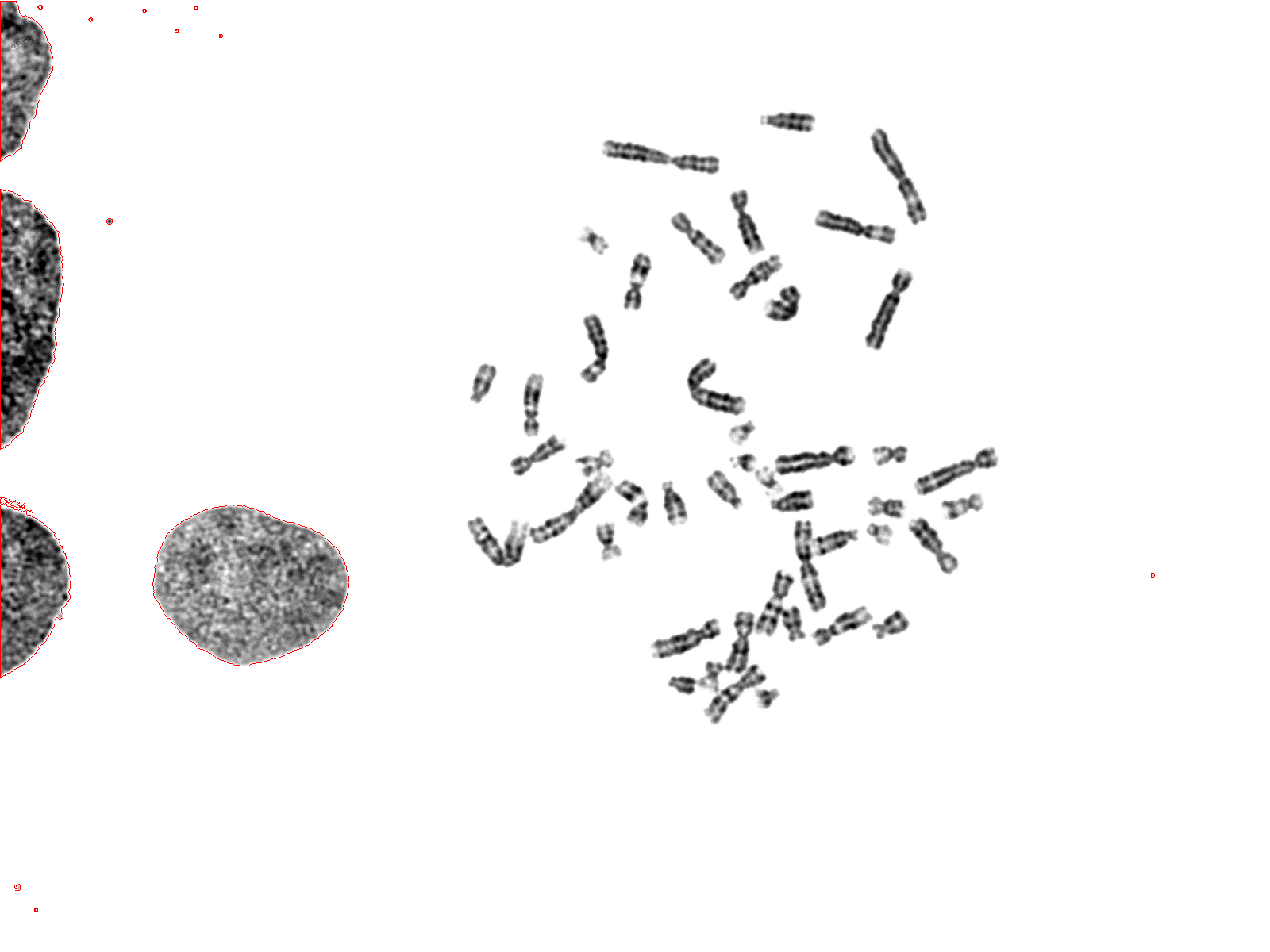

Supplement: Supplementary file 1 [file DataSheet1.ZIP › Supplementary Materials/Figure2 karyotyping in II 2.tif]

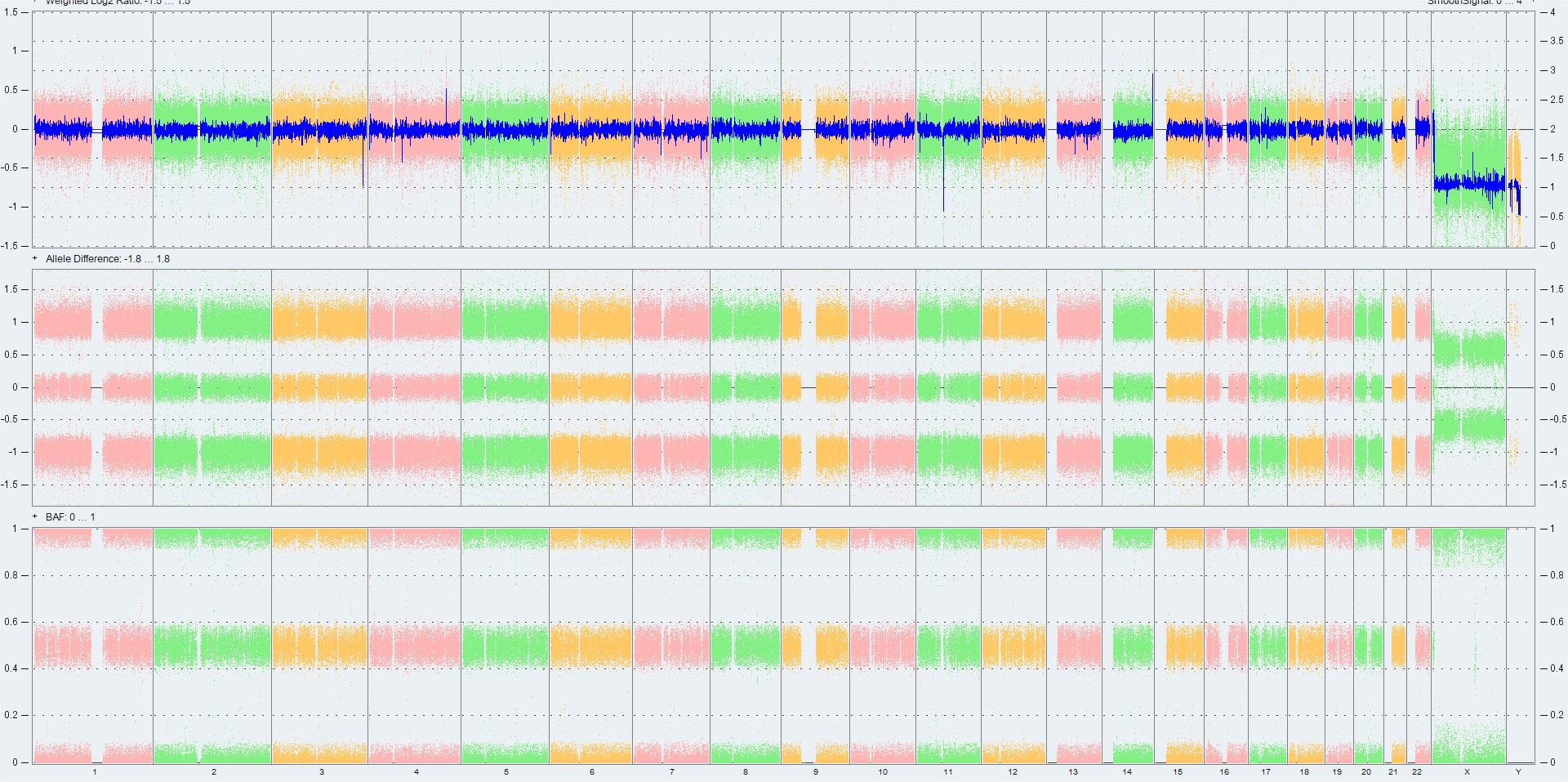

Supplement: Supplementary file 1 [file DataSheet1.ZIP › Supplementary Materials/Figure3 CMA in II4.png]

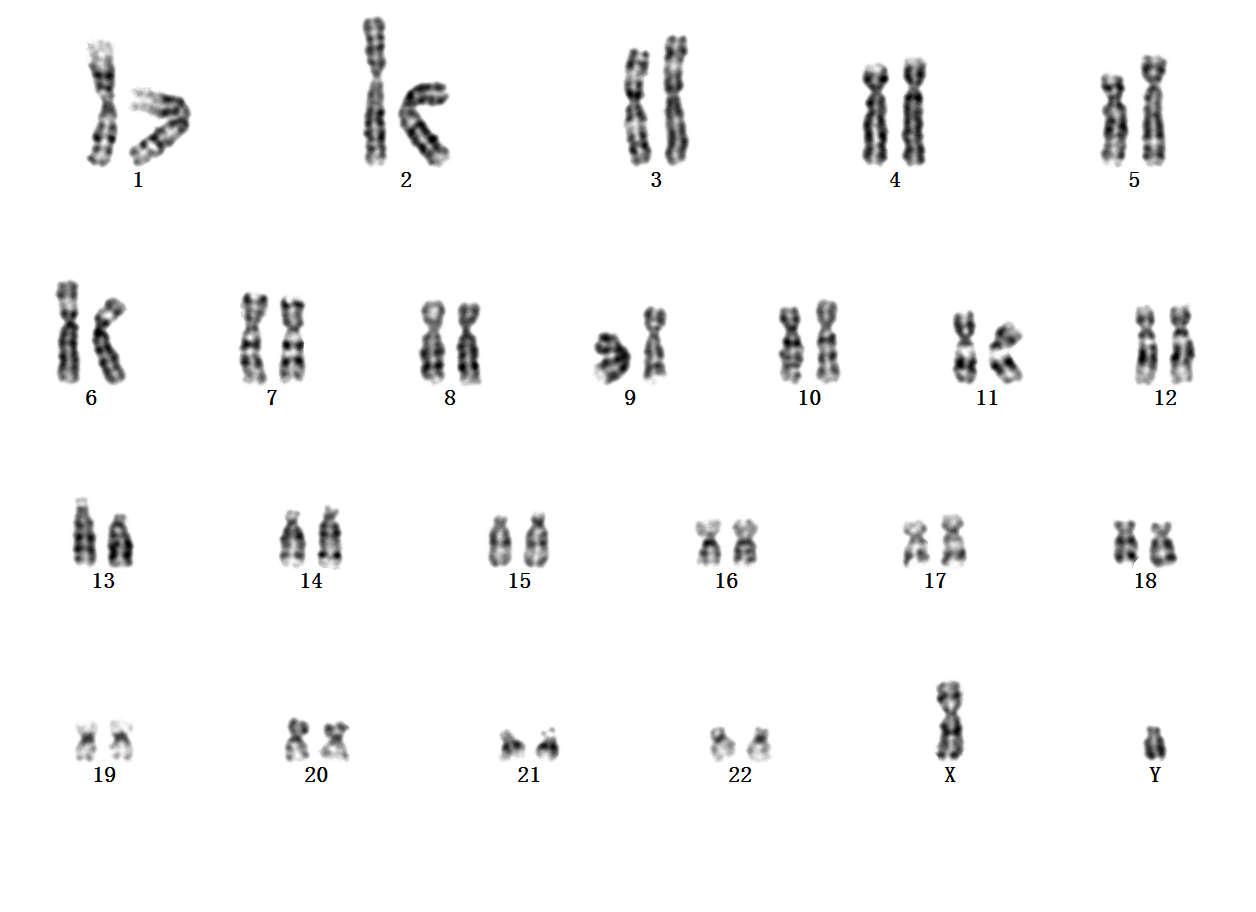

Supplement: Supplementary file 1 [file DataSheet1.ZIP › Supplementary Materials/Figure4 karyotyping in II 4.tif]
